# Phenotypic Diversity Analysis, Comprehensive Evaluation, and GWAS of Protein and Sucrose Content in Guizhou Local Peanut Germplasm Based on Three Consecutive Years of Phenotypic Data

**DOI:** 10.3390/genes17101164

**Published:** 2026-09-22

**Authors:** Qinglin Rao, Liangqiang Cheng, Min Jiang, Jianwei Lv, Tinghui Hu, Jinhua Wang, Jianqin Zhang, Jun Wang

**Affiliations:** Guizhou Institute of Oil Crops, Guizhou Academy of Agricultural Sciences, Guiyang 550006, China; raoqinglin89@163.com (Q.R.);

**Keywords:** peanut, germplasm resources, phenotypic diversity, comprehensive evaluation, GWAS, protein, sucrose

## Abstract

Background/Objectives: Peanut landraces (*Arachis hypogaea* L.) from Guizhou, a karst region of southwestern China, represent an underexploited breeding resource whose phenotypic architecture and marker associations remain insufficiently characterized. Methods: We evaluated 300 local accessions for 30 morphological, agronomic, and quality traits using three-year mean values and analyzed the data using phenotypic diversity statistics, Pearson correlation, stepwise regression, principal component analysis (PCA), hierarchical clustering, and genome-wide association study (GWAS). Results: The collection exhibited substantial phenotypic variation, with Shannon-Wiener indices ranging from 0.54 for leaf color to 2.09 for the equivalent yield per hectare (fresh weight). The Shannon-Wiener index for seedling uniformity was 0.69 in both Liupanshui and Tongren, indicating comparable phenotypic diversity for this trait rather than a direct measure of seedling uniformity. Guiyang accessions had the highest Shannon-Wiener index for sucrose content (1.99). Among 465 trait pairs, 113 showed significant positive correlations and 76 showed significant negative correlations. Stepwise regression retained 100-kernel weight, leaf color, leaf shape, 100-pod weight, main stem height, length of the first branches, and pod number per plant as the main statistical predictors of quality traits. PCA retained eight principal components with eigenvalues above one, which together explained 75.77% of the total variance. A total of 137 accessions had composite scores ≥ 0, and the top ten, including G23212 and G23172, were selected as candidate elite materials. Cluster analysis divided the accessions into four groups and identified 23 representative types. At a suggestive threshold of −log10(P) > 3, the GWAS detected 33 protein- and 27 sucrose-associated SNPs; these loci should be regarded as candidate associations pending validation. Conclusions: Collectively, these results indicate that Guizhou peanut landraces harbor substantial, geographically differentiated phenotypic variation and provide candidate materials and loci for further germplasm evaluation and marker validation.

## 1. Introduction

Peanut (*Arachis hypogaea* L.) is a dicotyledonous species of the genus Arachis Linn. in the family Leguminosae. Cultivated forms are allotetraploid (AABB, 2*n* = 4x = 40) and originated in the Brazil–Peru region of South America. With strong adaptability, peanut is widely cultivated worldwide and ranks among the world’s important oilseed and cash crops. The crop’s industry plays a central role in edible oil supply, dietary protein intake, food processing, and farmers’ incomes. Against a backdrop of global population growth and climate change, demand for high-yielding, high-quality, and stress-tolerant peanut cultivars is increasing. Guizhou Province, in southwestern China, has a complex, heterogeneous geography and diverse climates that create unique conditions for the evolution of peanut germplasm and support rich crop genetic diversity. However, the value of germplasm from this region has not yet been fully exploited. Peanut kernels are rich in fat and protein, making them an important raw material for edible oils, plant-derived proteins, and food processing. Mature peanut kernels contain approximately 25% protein [1], but their amino acid profile still requires improvement [2]. Peanut germplasm shows substantial variation in fat and protein content, carbon source, and fatty acid composition. Quality trait evaluation can identify high-oil, high-protein, and high-sugar lines [2,3]. Carbon source and sugar content are used to distinguish fresh from roasted peanuts and similar product types [4,5]. Oleic acid content and the oleic/linoleic acid ratio serve as criteria for selecting high-oleic germplasm [6].

The development and use of germplasm resources underpin peanut genetic improvement [7]. Analyzing their genetic composition and relationships is essential for breeding new cultivated varieties [8]. Local varieties (landraces), which result from long-term natural and human selection, retain high genetic diversity and are indispensable for stress resistance, regional adaptation, and unique quality traits [9,10]. Therefore, systematically collecting, conserving, and evaluating local germplasm is essential for maintaining crop genetic diversity and supporting modern breeding programs.

Phenotypic diversity analysis is an effective approach for revealing the breeding potential of germplasm resources [11,12]. Evaluating agronomic and quality traits is crucial for identifying candidate parents [3]. Systematic assessment of key traits enables selection of germplasm with desirable characteristics for targeted trait improvement, whereas cluster analysis of local varieties can reveal phenotypic structure and geographic patterns that inform conservation and utilization strategies [10,13]. Many researchers now use SSR markers, QTL mapping, and GWAS to accelerate breeding [14,15]; however, these approaches require robust phenotypic data [16,17]. We therefore performed phenotypic analyses over three consecutive years. Multi-year data can reduce environmental noise and improve the robustness of association analyses, but they cannot eliminate environment-induced false associations. We also examined trait associations to identify statistical predictors of quality traits rather than to establish causal chains among traits [18,19]. Yin et al. (2024), using approximately 300 rice germplasms and combining phenotypic measurements with the GWAS, identified a new grain-width QTL [20]. Tripathy (2020) screened over 200 rice germplasms and detected significant variation in Fe and Zn contents, demonstrating that phenotypic screening can directly support micronutrient breeding [21]. Zhu and Wang (2025) systematically evaluated phenotypic diversity in fresh maize germplasm and found pronounced variation in quality traits among Chinese accessions [22]. Cheng and Wang (2025) analyzed global fresh-maize germplasm data and revealed a lack of correspondence between geographic origin and phenotypic clustering, underscoring the need for systematic phenotypic data collection [23]. Banla et al. (2020) conducted a phenotypic diversity analysis of Togo peanut cultivars and found that local germplasm exhibited greater variation in oleic acid and protein than introduced varieties, suggesting that local germplasm has value for quality improvement [24]. Abady et al. (2021) assessed genetic diversity and population structure in peanut germplasm by combining phenotypic traits with SNP markers and identified unique allele combinations in drought-resistant lines [8]. Qureshi et al. (2023) evaluated quality traits in 21 sesame germplasms and reported differences in oil content and fatty acid composition [25]. Dalamu et al. (2023) conducted SSR marker and phenotypic trait analyses of native Indian potato germplasm and found a weak correlation between phenotypic clustering and geographical origin [26]. These findings collectively indicate that phenotypic characterization is a prerequisite for efficient utilization of crop germplasm resources.

The availability of peanut reference genomes and high-density molecular markers provides strong support for GWAS analyses of the genetic basis of yield and quality traits [27,28]. To date, multiple QTLs and candidate genes significantly associated with yield, protein, sucrose, fatty acids, and flavor have been identified in peanut, and corresponding molecular markers have been developed [29,30,31]. Marker-assisted selection for traits such as high oleic acid has progressed substantially and has been applied successfully to improve high-oleic peanut varieties [32,33]. However, GWAS reliability depends heavily on high-quality, multi-year phenotypic data [19]. Combining multi-year phenotypic evaluation with association analysis can reduce environmental noise and improve the stability of candidate loci [29,30].

Accessions were collected during the Third National Crop Germplasm Resources Census (2017–2022); phenotypic evaluation was conducted in three consecutive years from 2022 to 2024 at the same experimental site. This study analyzed phenotypic diversity and performed a comprehensive evaluation of growth period, plant, yield, and quality traits, and conducted the GWAS for seed protein and sucrose contents in the 300 local accessions. We (1) used the Shannon-Wiener diversity index (H’) and coefficient of variation to describe phenotypic variation across accessions and collection regions; (2) used the Pearson correlation and stepwise regression to identify statistical associations and predictors of quality traits; (3) used PCA and cluster analysis to identify candidate parental materials; and (4) performed the GWAS to identify candidate SNP associations for protein and sucrose. These analyses provide a foundation for germplasm evaluation, parental selection, and subsequent marker validation in Guizhou peanut breeding.

## 2. Materials and Methods

Field experiments were conducted in 2022, 2023, and 2024 at the peanut experimental base of the Guizhou Academy of Agricultural Sciences (Huaxi District, Guiyang City, Guizhou Province, China). Each year, the 300 accessions were planted in a germplasm observation nursery. Owing to the resource-maintenance layout, plots were not randomized or replicated within a year; each accession was grown in four consecutive rows 2.5 m long with 0.40 m row spacing and 0.20 m hill spacing, and conventional field management was applied uniformly. Five representative plants per accession were sampled as measurement replicates. During the seedling stage, seedling uniformity was recorded; sowing and maturity dates were recorded to calculate the days from sowing to maturity; resistance to pests and diseases, leaf shape, and leaf color were scored during the pegging stage. Flowering dates were recorded when 10% and 90% of plants had flowers, and the flowering period was calculated as the interval between these dates.

### 2.1. Test Materials

Three hundred local peanut germplasm accessions from Guizhou were distributed across nine prefectures and cities: Guiyang, Liupanshui, Zunyi, Tongren, Southwest Guizhou Autonomous Prefecture, Bijie, Anshun, Qiandongnan Miao and Dong Autonomous Prefecture, and Qiannan Buyi and Miao Autonomous Prefecture. All accessions were collected during the Third National Crop Germplasm Resources Census (2017–2022) and represent farmers’ retained seed sources. Field phenotyping was conducted from 2022 to 2024; therefore, the collection period and the phenotypic evaluation period are distinct.

The experimental site was at the Guizhou Academy of Agricultural Sciences in Huaxi District, Guiyang City, Guizhou Province, which lies in a humid subtropical plateau monsoon climate. Annual mean temperatures range from 4.7 to 14.9 °C and 14.9 to 23.3 °C.

### 2.2. Experimental Methods

#### 2.2.1. Recording of Physical Characteristics

Phenotypic recording was conducted in accordance with the *Descriptors and Data Standard for Peanut (Arachis* spp.*) (Jiang Huifang)*. Days from sowing to maturity: the number of days from sowing to physiological maturity; flowering period: the number of days from first flowering to last flowering. Quantitative traits were measured using a ruler: height of main stem and length of first branches; total branching number, number of branches with pods, and pod number per plant were counted manually according to actual conditions, repeated five times; ACS-30C6 electronic pricing scale (manufacturer and city not recorded in the original laboratory records) was used to weigh fresh fruits in the community. Equivalent yield per hectare (fresh weight), 100-pod weight, 100-kernel weight, 100-seed weight, kernel recovery rate, and hull percentage were weighed using an LQ-A6002 electronic balance (manufacturer and city not recorded in the original laboratory records) and calculated thereafter. Fat, protein, palmitic, oleic acid, linoleic acid, aspartic acid, glutamic acid, arginine, source, total sugar, and fructose were determined using a NIRS DS2500 near-infrared multifunctional quality analyzer (FOSS Analytical, Hillerød, Denmark) at the Guizhou Oil Crops Research Institute after the seeds harvested that year were air-dried naturally for 3 months. The ratio of oleic acid to linoleic acid was calculated from the values of oleic acid and linoleic acid.

Whole-genome resequencing was performed using paired-end reads on the Illumina NovaSeq platform. After read alignment and variant calling, only biallelic SNPs were retained if they had a call rate ≥ 90%, a minor allele frequency (MAF) > 0.05, and no evidence of excessive heterozygosity. A total of 17,922 high-quality SNPs passed these filters. The GWAS was performed with EMMAX using a variance-component mixed model; the genomic relationship (kinship) matrix was constructed from the filtered SNPs using the identity-by-state method, and no additional principal-component covariates were fitted in the final model. Because no formal multiple-testing correction was applied, −log10(P) > 3 was used as a suggestive threshold; the Bonferroni threshold for alpha = 0.05 across 17,922 SNPs was −log10(P) = 5.55. QQ plots were inspected, and the genomic inflation factors were approximately 1.0 for both traits (see Section 3.5).

#### 2.2.2. Phenotypic Trait Scoring

Phenotypic trait values during the reproductive period are detailed in Table 1.

#### 2.2.3. Data Analysis

Trait frequencies, means, standard deviations, coefficients of variation, and Shannon-Wiener diversity indices were calculated using WPS Excel (version not recorded in the original analysis records). Distribution maps were generated using Origin 2024 (OriginLab Corporation, Northampton, MA, USA). Continuous traits were classified into 10 levels based on the mean and standard deviation, and H’ was calculated from the resulting frequencies. For all subsequent analyses, the three-year mean of each trait for each accession was used; each yearly value was first calculated from five sampled plants. Pearson correlation coefficients were calculated among the 30 traits using two-sided tests; *p* < 0.05 was considered significant and *p* < 0.01 highly significant. Stepwise regression was performed with quality traits as dependent variables and growth period, plant, and yield traits as candidate predictors, using an entry threshold of *p* < 0.05 and a removal threshold of *p* > 0.10. Adjusted R2, standardized regression coefficients, variance inflation factors (VIFs), Durbin–Watson statistics, Breusch–Pagan tests, and Jarque–Bera tests were used to evaluate model fit and residual diagnostics (Table S1). PCA and hierarchical clustering (Euclidean distance) were performed as described in the Results. The GWAS and significance-threshold details are provided in Section 2.2.1.

## 3. Results

### 3.1. Phenotypic Diversity of 300 Local Peanut (A. hypogaea) Germplasm Resources from Guizhou

Phenotypic traits across the peanut growth cycle and kernel quality affect yield and downstream applications; therefore, growth-stage, plant, yield, and quality traits were analyzed at two levels: the entire collection and the collection region. Growth-period traits included seedling uniformity, flowering period, days from sowing to maturity, resistance to pests and diseases, leaflet shape, and leaflet color. Plant traits included height of the main stem, length of the first branches, total branching number, number of branches with pods, pod number per plant, and pod yield per plant. Yield-related traits included equivalent yield per hectare (fresh weight), 100-pod weight, 100-kernel weight, 100-seed weight, kernel recovery rate, and hull percentage. Quality traits included fat, protein, palmitic acid, oleic acid, linoleic acid, oleic/linoleic acid ratio, aspartic acid, glutamic acid, arginine, sucrose, total sugar, and fructose.

#### 3.1.1. Phenotypic Diversity of Traits During the Reproductive Period

Phenological traits in the peanut germplasm showed variable frequency distributions, with phenotypic diversity indices ranging from 0.68 to 1.31; only the flowering period exceeded a diversity index of 1 (Table 2). Among the six traits, flowering period was the most differentiated (seven categories) and had the highest diversity index (1.31). Leaflet shape followed with six categories and a diversity index of 0.98. Seedling uniformity was the least differentiated, having only two categories and a phenotypic diversity index of 0.68.

Phenotypic diversity indices were calculated separately for each collection region. Qiannan Buyi and Miao Autonomous Prefecture and Bijie showed lower values for all six reproductive-stage traits than that of the other regions, with Bijie showing particularly reduced phenotypic diversity. The diversity indices of Southwest Guizhou Autonomous Prefecture and Qiandongnan Miao and Dong Autonomous Prefecture were similar, although Southwest Guizhou had the higher value. For seedling uniformity, H’ was 0.69 in both Liupanshui and Tongren, indicating comparable phenotypic diversity rather than a direct measure of seedling uniformity. In Liupanshui, no pale-green leaflet color was observed, and leaflet shape contributed most to the higher diversity index in Southwest Guizhou Autonomous Prefecture, indicating greater phenotypic variation in leaflet morphology in that region.

#### 3.1.2. Phenotypic Diversity of Plant Phenotypic Traits

The mean phenotypic diversity index for six plant traits across nine prefectures and cities in Guizhou was 1.98. The highest index occurred for the number of branches with pods (2.01), while length of first branches and pod yield per plant showed the lowest values (1.96). Local germplasm from Zunyi City exhibited a notably higher diversity index. The mean coefficient of variation for the plant traits was 23.12%, indicating substantial genetic variability. Distributions and coefficients of variation for the six quantitative traits, including height of main stem and length of first branches, for all tested accessions are shown in Figure 1 and Table 3.

The number of secondary branches was approximately normally distributed, with a coefficient of variation of 20.68% and a mean of 5.91 ± 1.22. Height of the main stem, length of the first branches, total branching number, pod number per plant, and pod yield per plant were also approximately normally distributed, with relatively concentrated values and low dispersion. The coefficients of variation for the length of the first branches and total branching number were 18.91% and 17.86%, respectively, with means of 40.33 ± 7.63 and 6.16 ± 1.10. Phenotypic diversity indices for the six traits were comparable, ranging from 1.96 to 2.01; total branching number and number of branches with pods were 2.00 and 2.01, respectively. These indices describe the extent and dispersion of phenotypic variation in this collection; they do not by themselves demonstrate the absence or presence of artificial selection, because environmental effects, sampling, and breeding history can also influence trait distributions.

The coefficients of variation (CVs) for peanut traits differed among collection locations (Table 3). Total branching number showed the smallest CVs, ranging from 8.87% to 22.16%. Pod yield per plant exhibited the largest CVs across locations, ranging from 21.36% to 52.88%. The CVs for length of the first branches, total branching number, and number of branches with pods remained below 25% across locations, with mean CVs of 17.65%, 15.67%, and 18.65%, respectively, indicating limited phenotypic variation for these traits. Pod yield per plant varied markedly among locations: all nine prefectures/cities had CVs above 20%, with the smallest in Liupanshui (24.70%) and the largest in Guiyang (52.88%). Guiyang and Qiannan had pod yield per plant CVs exceeding 30%. Pod number per plant also varied, from 15.67% to 41.98%, with Guiyang, Tongren, and Bijie showing CVs above 30%. These patterns indicate that local cultivars from Guiyang, Tongren, Bijie, and Qiannan have relatively high per-plant yield variability and greater among-accession differences.

#### 3.1.3. Phenotypic Diversity of Yield-Related Traits

Across nine prefectures/cities, the mean diversity index for six yield-related traits was 1.98, and the average coefficient of variation was 21.78%, indicating substantial genetic diversity. Distributions, coefficients of variation, and diversity indices for all accessions for the six yield traits—equivalent yield per hectare (fresh weight), 100-pod weight, 100-kernel weight, 100-seed weight, kernel recovery rate, and hull percentage—are shown in Figure 2 and Table 3. Equivalent yield per hectare (fresh weight), 100-seed weight, kernel recovery rate, and hull percentage followed approximately normal distributions, with coefficients of variation of 37.43%, 19.04%, 13.96%, and 24.07%, respectively, and means ± SD of 317.86 ± 118.98, 54.29 ± 10.34, 63.29 ± 8.83, and 36.71 ± 8.83. The remaining yield variables were also approximately normally distributed; 100-pod weight and 100-kernel weight were primarily distributed in the ranges 101.49–184.16 g and 60.32–125.37 g, respectively. In addition, the phenotypic diversity indices for the six yield traits were similar, ranging from 1.92 to 2.09; notably, kernel recovery rate and hull percentage had identical diversity indices. These results suggest that the six yield-related traits have not been substantially affected by human intervention.

Coefficients of variation for the same yield-related traits differed among sources (see Table 3). Kernel recovery rate showed the smallest variation across sources, ranging from 9.78% to 16.35%, all below 17%, which indicates limited variability. The coefficient of variation for 100-kernel weight was also relatively low, with a mean of 19.39%, indicating that these two traits had comparatively low variability. Equivalent yield per hectare (fresh weight) displayed the largest variation, ranging from 20.27% to 48.70%. The magnitudes of variation for 100-pod weight, 100-seed weight, and hull percentage were similar, ranging from 10.52% to 16.45%. For local resources from Guiyang city, coefficients of variation were relatively high for 100-pod weight, 100-kernel weight, 100-seed weight, kernel recovery rate, and hull percentage, whereas those from Bijie city were relatively low. Equivalent yield per hectare (fresh weight) showed a relatively high coefficient of variation for materials from Qiandongnan Miao and Dong Autonomous Prefecture and a lower coefficient for Liupanshui city. This pattern indirectly indicates that yield information is relatively rich for varieties from Guiyang city and Qiandongnan Miao and Dong Autonomous Prefecture, producing greater differences in the equivalent yield per hectare (fresh weight) among varieties.

#### 3.1.4. Phenotypic Diversity of Quality Traits

The twelve quality traits of peanut germplasm from different collection regions showed a mean phenotypic diversity index of 1.96 and a mean coefficient of variation of 6.10%, indicating relatively modest phenotypic variation for these traits. Figure 3 and Table 3 present the distributions, CVs, and diversity indices for all twelve traits. Fat, protein, palmitic acid, oleic acid, arginine, aspartic acid, sucrose, fructose, and total sugar were approximately normally distributed; their diversity indices were similar (1.92–2.07), and their CVs ranged from 0.99% to 9.18%. Protein and fructose had CVs of 7.09% and 7.71%, with means of 25.51 ± 1.81% and 3.10 ± 0.24%, respectively. Palmitic acid and fructose showed relatively concentrated distributions (10.47–12.47% and 2.64–3.48%, respectively). Oleic acid and the oleic/linoleic acid ratio had similar diversity indices (1.84 and 1.81). The remaining three traits had H’ values between 1.81 and 2.01 and CVs up to 13.85%. These patterns indicate limited phenotypic spread in several quality traits; they should not be interpreted as direct evidence of selection without genomic and historical data.

The coefficients of variation (CVs) for the same peanut quality traits differed among source locations (Table 3). Total sugar showed the smallest inter-location CV, ranging from 0.71% to 1.05%. Fat, protein, palmitic acid, linoleic acid, aspartic acid, glutamic acid, and arginine had relatively small inter-location CVs, with mean CVs between 2.69% and 3.12%, indicating limited variability for these traits. The oleic acid to linoleic acid ratio exhibited the greatest variability, with CVs of 9.40% to 21.04%. Oleic acid, source, and fructose also showed relatively large CV differences across locations. Specifically, Guiyang City had higher CVs for fat and fructose; Southwest Guizhou Autonomous Prefecture had higher CVs for palmitic acid, oleic acid, linoleic acid, the oleic/linoleic ratio, and glutamic acid; Bijie City had a higher CV for aspartic acid; Anshun had a higher CV for arginine; and Qiandongnan Miao and Dong Autonomous Prefecture had higher CVs for protein and source. These results indicate substantial variation among varieties.

### 3.2. Correlation Analysis Among Phenotypic Traits of Local Peanut Germplasm Resources in Guizhou

#### 3.2.1. Correlation Analysis Among Phenotypic Traits

Across the 465 pairwise correlations computed among 30 agronomic and quality-related phenotypic traits, 113 pairs showed significant positive correlations and 76 pairs showed significant negative correlations (Figure 4); *p* < 0.05 was considered significant and *p* < 0.01 highly significant. Negative statistical associations were observed between days from sowing to maturity and sucrose/fructose; leaflet shape and palmitic/linoleic acid; leaflet color and protein/palmitic acid; and between arginine and several plant and yield traits. Positive statistical associations were observed between leaflet shape/color and oleic acid or the oleic/linoleic ratio; between plant height or branching traits and sucrose/fructose; and between total sugar and 100-pod weight, 100-kernel weight, and kernel recovery rate. These correlation patterns can guide trait selection, but they do not establish causal promoting or inhibitory relationships among traits.

#### 3.2.2. Screening of Influential Factors and Establishment of Regression Equations for Quality Traits by Using Whole-Life-Stage Traits and Agronomic Traits

Because many traits were correlated, stepwise regression was used to examine statistical predictors of quality traits. Table 4 lists the final predictors and equations, and Table S1 reports adjusted R2, standardized coefficients, VIFs, and residual diagnostics. Across the fitted models, adjusted R2 ranged from 0.037 to 0.143, and VIFs were below 1.5. Fat content was positively associated with 100-kernel weight; protein content was negatively associated with leaf color; and palmitic acid was negatively associated with leaf color but positively associated with 100-seed weight. Oleic acid, linoleic acid, and their ratio were associated with leaf shape and 100-seed weight. Glutamic acid was associated with hull percentage and resistance to pests and diseases, and arginine was associated with length of the first branches and 100-pod weight. Sucrose was associated with main stem height, pod number per plant, leaf shape, 100-pod weight, and pod yield per plant. Total sugar was associated with leaf shape and 100-pod weight, whereas fructose was associated with 100-pod weight, leaf shape, and main stem height. No significant predictor was retained for aspartic acid. These variables should be regarded as statistical predictors in this population, not demonstrated determinants of the quality traits.

### 3.3. Comprehensive Evaluation of Phenotypic Traits of Local Peanut Germplasm Resources in Guizhou

We performed principal component analysis of phenotypic traits of Guizhou local peanut germplasm using DPS 25.0. Eight principal components had eigenvalues greater than 1. As shown in Table 5, the cumulative variance contribution reached 75.77%, indicating that these components capture over 75% of the phenotypic variation in the Guizhou germplasm. PC1 and PC6 were mainly represented by number of branches with pods (0.29 and 0.33), total branching number (0.28 and 0.31), kernel recovery rate (0.28), and pod number per plant (0.34), and were therefore summarized as yield-related factors. PC2 was dominated by linoleic acid (0.32) and protein (0.30) and was summarized as a protein–linoleic acid synergistic factor; PC3 loaded primarily on 100-kernel weight (0.45), 100-pod weight (0.42), and 100-seed weight (0.36), and was therefore labeled the kernel factor. PC4 was dominated by fructose (0.39), source (0.36), and total sugar (0.25), and was labeled the sugar factor. PC5 was characterized mainly by arginine (0.31), seedling uniformity (0.28), and glutamic acid (0.28), and was labeled the amino-acid factor. PC7 and PC8 were dominated, respectively, by days from sowing to maturity (0.56), fructose (0.32), and pod number per plant (0.26), and by days from sowing to maturity (0.28), length of first branches (0.27), and height of main stem (0.26); both were therefore summarized as phenological-duration factors. These results indicate that, among Guizhou local peanut germplasm resources, fruiting-branch number, pods /plant, 100-kernel weight, fructose content, arginine content, and overall growth duration show relatively large variation.

The composite scores of 300 test materials were calculated using principal component analysis. Thirty phenotypic traits are denoted by X_1_-X_30_, as defined by the following equation:

F_1_ = 0.15X_1_ − 0.14X_2_ + 0.18X_3_ + 0.06X_4_ + 0.08X_5_ − 0.14X_6_ + 0.22X_7_ + 0.24X_8_ + 0.28X_9_ + 0.29X_10_ + 0.20X_11_ − 0.23X_12_ + 0.20X_13_ − 0.02X_14_ + 0.17X_15_ − 0.07X_16_ + 0.28X_17_ − 0.28X_18_ + 0.09X_19_ − 0.20X_20_ − 0.13X_21_ + 0.20X_22_ − 0.20X_23_ + 0.20X_24_ − 0.21X_25_ − 0.19X_26_ − 0.15X_27_ + 0.07X_28_ + 0.13X_29_ + 0.08X_30_

F_2_ = 0.00X_1_ − 0.15X_2_ + 0.07X_3_ − 0.08X_4_ − 0.09X_5_ − 0.03X_6_ + 0.23X_7_ + 0.23X_8_ + 0.18X_9_ + 0.20X_10_ + 0.13X_11_ − 0.16X_12_ + 0.17X_13_ + 0.01X_14_ + 0.04X_15_ + 0.01X_16_ + 0.07X_17_ − 0.07X_18_ − 0.16X_19_ + 0.30X_20_ + 0.26X_21_ − 0.34X_22_ + 0.32X_23_ − 0.34X_24_ + 0.25X_25_ + 0.24X_26_ + 0.07X_27_ + 0.19X_28_ + 0.07X_29_ + 0.09X_30_

F_3_ = 0.06X_1_ + 0.00X_2_ + 0.06X_3_ + 0.29X_4_ + 0.34X_5_ + 0.03X_6_ − 0.14X_7_ − 0.13X_8_ − 0.06X_9_ − 0.05X_10_ − 0.19X_11_ − 0.02X_12_ + 0.14X_13_ + 0.42X_14_ + 0.45X_15_ + 0.36X_16_ + 0.17X_17_ − 0.17X_18_ + 0.10X_19_ + 0.01X_20_ − 0.01X_21_ − 0.06X_22_ + 0.13X_23_ − 0.08X_24_ + 0.10X_25_ + 0.00X_26_ + 0.19X_27_ − 0.13X_28_ + 0.11X_29_ − 0.14X_30_

F_4_ = −0.23X_1_ − 0.10X_2_ − 0.24X_3_ + 0.23X_4_ + 0.10X_5_ + 0.18X_6_ + 0.07X_7_ + 0.04X_8_ + 0.00X_9_ − 0.01X_10_ − 0.01X_11_ − 0.11X_12_ + 0.17X_13_ + 0.13X_14_ − 0.15X_15_ + 0.21X_16_ − 0.23X_17_ + 0.23X_18_ − 0.27X_19_ + 0.03X_20_ − 0.16X_21_ + 0.19X_22_ − 0.19X_23_ + 0.18X_24_ + 0.09X_25_ + 0.10X_26_ + 0.08X_27_ + 0.36X_28_ + 0.25X_29_ + 0.39X_30_

F_5_ = 0.28X_1_ + 0.06X_2_ + 0.26X_3_ + 0.09X_4_ + 0.18X_5_ − 0.15X_6_ − 0.17X_7_ − 0.17X_8_ − 0.13X_9_ − 0.06X_10_ + 0.08X_11_ + 0.13X_12_ − 0.25X_13_ − 0.20X_14_ − 0.01X_15_ − 0.26X_16_ + 0.20X_17_ − 0.20X_18_ − 0.29X_19_ + 0.09X_20_ − 0.21X_21_ + 0.08X_22_ − 0.09X_23_ + 0.09X_24_ + 0.26X_25_ + 0.28X_26_ + 0.31X_27_ + 0.15X_28_ + 0.08X_29_ + 0.16X_30_

F_6_ = 0.08X_1_ + 0.09X_2_ − 0.07X_3_ + 0.09X_4_ + 0.16X_5_ + 0.12X_6_ + 0.09X_7_ + 0.15X_8_ + 0.31X_9_ + 0.33X_10_ + 0.34X_11_ + 0.20X_12_ − 0.12X_13_ + 0.15X_14_ + 0.00X_15_ + 0.07X_16_ − 0.20X_17_ + 0.20X_18_ + 0.09X_19_ + 0.08X_20_ − 0.01X_21_ + 0.13X_22_ − 0.14X_23_ + 0.16X_24_ + 0.21X_25_ + 0.18X_26_ + 0.19X_27_ − 0.16X_28_ − 0.35X_29_ − 0.28X_30_

F_7_ = 0.00X_1_ + 0.56X_2_ − 0.01X_3_ + 0.18X_4_ + 0.14X_5_ − 0.41X_6_ − 0.07X_7_ − 0.02X_8_ + 0.11X_9_ + 0.12X_10_ + 0.26X_11_ + 0.10X_12_ − 0.19X_13_ + 0.08X_14_ − 0.01X_15_ + 0.05X_16_ − 0.16X_17_ + 0.16X_18_ + 0.01X_19_ + 0.03X_20_ + 0.04X_21_ − 0.14X_22_ + 0.15X_23_ − 0.14X_24_ − 0.17X_25_ − 0.15X_26_ − 0.17X_27_ + 0.07X_28_ + 0.32X_29_ + 0.13X_30_ (1)

F_8_ = −0.09X_1_ + 0.28X_2_ + 0.25X_3_ − 0.24X_4_ − 0.19X_5_ − 0.26X_6_ + 0.26X_7_ + 0.27X_8_ − 0.27X_9_ − 0.23X_10_ − 0.07X_11_ + 0.08X12 − 0.07X_13_ + 0.26X_14_ + 0.21X_15_ + 0.11X_16_ + 0.02X_17_ − 0.02X_18_ + 0.15X_19_ + 0.10X_20_ + 0.16X_21_ + 0.20X_22_ − 0.15X_23_ + 0.18X_24_ + 0.08X_25_ + 0.13X_26_ + 0.03X_27_ + 0.28X_28_ − 0.14X_29_ + 0.04X_30_

F = 0.25F_1_ + 0.20F_2_ + 0.15F_3_ + 0.13F_4_ + 0.12F_5_ + 0.06F_6_ + 0.05F_7_ + 0.05F_8_ (2)

The comprehensive score F was calculated from the equations above (Table 6) and used to evaluate overall performance. A total of 137 accessions with F ≥ 0.00 were classified as having above-average overall phenotypic performance, and 34 accessions with F ≥ 1.00 were designated as priority candidates. The ten highest-ranked accessions were G23212 (Songtao Chengguan town peanut, F = 4.05), G23169 (Xieqiao peanut, F = 2.60), G23186 (Pomei multi-seed variety, F = 2.06), G23289 (Guihua 169, F = 1.95), G23253 (Chishui Wanglongzhen-2, F = 1.93), G23294 (Guangshun red-skinned, F = 1.72), G23131 (Puding peanut, F = 1.63), G23109 (Bianyang peanut, F = 1.51), G23174 (Xingyi small peanut, F = 1.48), and G23172 (Congjiang big peanut, F = 1.36). These lines can serve as candidate parental materials for peanut breeding, but their performance requires multi-environment validation.

### 3.4. Cluster Analysis of Local Peanut Germplasm Resources in Guizhou and Characteristics of Each Group

To clarify phenotypic variation among local peanut germplasm resources in Guizhou and to provide a theoretical basis for their effective use—particularly for parental selection in breeding—we performed cluster analysis on plant, yield, and quality traits surveyed continuously over three years. The clustering results showed that the 300 local Guizhou peanut germplasm accessions were classified into four groups (Figure 5). The four clusters (I–IV) differ in sample size: Cluster I contained 105 samples (the largest), Cluster II contained 47 samples (the smallest), Cluster III contained 54 samples, and Cluster IV contained 94 samples. Each cluster included local varieties from at least eight distinct source locations. Guiyang City, Buyi and Miao Autonomous Prefecture of Qiannan, and Southwest Guizhou Autonomous Prefecture were largely concentrated in Cluster I, whereas Zunyi City dispersed relatively evenly across clusters. The remaining six prefectures and cities were scattered among the four clusters, and the local germplasm of Liupanshui City was absent from Cluster III. These results indicated that clustering did not strictly follow geographic origin and that each region had developed its own characteristic varieties.

We compared days from sowing to maturity, plant architecture, yield, and quality traits within each group (Table 7). Group I had the longest time of days from sowing to maturity and the smallest coefficient of variation for that trait. Group II showed the greatest seedling uniformity (smallest coefficient of variation) and performed strongly for time from sowing to maturity and pest and disease resistance. Group III was characterized mainly by wide-elliptic leaves, while Group IV exhibited the longest flowering period; Group I had a significantly higher pod yield per plant. Group II had significantly greater height of main stem, length of first branches, total branching number, number of branches with pods, pod number per plant, and equivalent yield per hectare (fresh weight). Group III had significantly higher 100-pod weight, 100-kernel weight, and kernel recovery rate. Group IV had significantly higher 100-seed weight and hull percentage compared with the other groups. Qualitative traits show that group I is characterized mainly by protein, linoleic acid, aspartic acid, glutamic acid, and arginine; group II by source and fructose; group III by fat, oleic acid, the ratio of oleic acid to linoleic acid, and total sugar; and group IV by palmitic acid. These findings imply that accessions in group I were likely constrained by insufficient caloric accumulation during peanut maturation, group II combined stable yield, high yield, good palatability, and earliness, group III constituted candidate materials for high-oleic varieties, and group IV could serve as parental candidates. Based on statistical analysis and clustering of 30 phenotypic traits in 300 peanut germplasm accessions, representative materials from each group were selected from the tested materials to provide references for future peanut cultivar improvement (Table 8).

### 3.5. Genome-Wide Association Analysis of Quality Traits

Using high-quality data comprising three consecutive years of protein and sucrose measurements for 300 Guizhou peanut germplasm accessions, we performed the GWAS with the EMMAX mixed model using 17,922 SNPs. The kinship matrix was estimated from the filtered SNPs using the identity-by-state method, and no additional principal-component covariates were included. Because −log10(P) > 3 is a suggestive threshold and the Bonferroni threshold for alpha = 0.05 was 5.55, the detected SNPs are reported as candidate/suggestive associations rather than genome-wide significant loci. For protein, 43 associated SNP loci were detected, 33 of which exceeded the suggestive threshold; these loci mapped to chromosomes 5, 9, 11, 12, 13, 14, 15, 16, and 20 (Figure 6). For sucrose, 41 associated SNP loci were detected, 27 of which exceeded the suggestive threshold; these loci mapped to chromosomes 11, 14, 15, 16, and 17 (Figure 7). The QQ plots were approximately consistent with the null distribution, with genomic inflation factors of approximately 1.0 for both traits. These loci provide candidate regions for subsequent validation, gene annotation, and marker development.

## 4. Discussion

We used 300 local peanut accessions from nine prefectures and cities in Guizhou, collected during the 2017–2022 Third National Crop Germplasm Resources Survey, as plant materials. Based on phenotypic evaluations conducted over three consecutive years, we assessed the phenotypic diversity for 30 traits spanning phenology, plant architecture, yield, and quality. The tested germplasm showed pronounced phenotypic variation for most traits, providing a valuable foundation for efficient breeding and sustainable development of the peanut industry [34]. We then used correlation analysis, stepwise regression, and PCA to identify statistical relationships and predictors of phenotypic traits, and combined composite scoring with cluster analysis to select superior lines and representative parents [34,35]. The GWAS for protein and sucrose provided candidate loci for subsequent validation and marker development [36,37].

### 4.1. Phenotypic Diversity and Breeding Value of Local Peanut Germplasm Resources in Guizhou

Analysis of traits during the reproductive period showed a Shannon-Wiener diversity index ranging from 0.68 to 1.31, with the flowering period having the highest value (1.31) and seedling uniformity the lowest (0.68). This pattern indicates substantial phenotypic variation in flowering time, consistent with previous studies of peanut germplasm (Hong et al., 2021 [38]; Upadhyaya et al., 2008 [39]), and supports selection of early- and late-maturing cultivars (Yol & Uzun, 2019 [40]; Daudi et al., 2021 [41]). Plant traits had a mean phenotypic diversity index of 1.98 and a mean coefficient of variation of 23.12%, indicating substantial phenotypic variation. The distribution of the number of secondary branches approximated normality, which is compatible with a complex trait but does not by itself establish polygenic control. Similarity among diversity indices indicates similar phenotypic diversity, not comparable genetic control, and low CVs may reflect limited phenotypic spread under the current environment rather than a conserved genetic basis or selective constraint. These results describe the phenotypic structure of the collection and identify traits with the potential for selection.

### 4.2. Source Differences and Regional Germplasm Utilization

Analysis of diversity indices by origin showed that Qiannan and Bijie had relatively low diversity throughout the reproductive period, whereas Southwest Guizhou and Qiandongnan had higher diversity, especially for leaflet shape. These differences may be associated with local climate, soil, management, or historical seed exchange, but the present study does not directly test these causes [42].

The coefficients of variation among landraces from different origins varied substantially. Pod yield per plant showed large variation in Guiyang and Qiannan, suggesting that these populations may be useful for selecting high-yielding individuals. In contrast, total branching number, length of the first branches, and number of branches bearing pods showed relatively low variation across origins; these patterns may reflect trait stability, environmental effects, or past selection, but they do not by themselves demonstrate strong selection pressure. Similar limited variation in cultivated peanut branching traits has been reported [42], and selection during domestication can reduce phenotypic variation in key agronomic traits [43]. Overall, the CV results indicate that several plant traits have relatively limited phenotypic spread in this collection.

Analysis of yield-related traits across origins indicated that local varieties from Guiyang and Qiandongnan contained more diverse yield-related phenotypes. For example, equivalent yield per hectare (fresh weight) had a relatively high coefficient of variation and may be useful for selecting candidate high-yielding accessions.

Marked differences in quality traits were observed among germplasm from different origins. Local varieties from Bijie had higher sucrose, total sugar, and fructose contents, making them promising candidates for fresh-eating peanut breeding. Local varieties from Guiyang and Qiannan had relatively high arginine levels and may serve as specialized germplasm. These results indicate geographic differences in quality traits, consistent with studies reporting variation in sugar-related traits and arginine content [44,45]. The causes of these differences remain to be tested in multi-environment trials.

Phenotypic differences among local cultivars from different collection regions may reflect ecological factors such as altitude, temperature, precipitation, and soil, as well as the farmers’ selection for yield and maturity. However, these mechanisms were not directly tested here. Holbrook and Isleib [42] noted that the distribution of peanut diversity is uneven across geographic origins, and germplasm from high-diversity regions may harbor useful allele combinations. Ren et al. [36] reported detectable genetic structure in major Chinese peanut cultivars. Consistent with Jarvis et al.’ [46] analysis of traditional varietal diversity, the highly diverse materials from southwestern and southeastern Guizhou may merit priority for conservation and further evaluation, but molecular data are needed to confirm genetic differentiation.

### 4.3. Trait Associations and Quality Improvement

Pearson correlation analyses revealed significant statistical associations among reproductive-period, agronomic, and quality traits. Fat content correlated positively with 100-pod and 100-kernel weights, whereas protein content correlated negatively with fat content and kernel recovery rate; these patterns may be compatible with lipid accumulation and protein–fat relationships but do not establish a metabolic trade-off [47]. Oleic acid correlated negatively with palmitic and linoleic acids, whereas the oleic/linoleic ratio correlated positively with several quality traits, consistent with the known regulation of fatty acid desaturation [32]. Arginine, sucrose, total sugar, and fructose showed significant associations with plant height, branching, pod number, pod yield, and seed size, indicating that plant architecture, developmental stage, and seed size covary with seed metabolites; these associations do not demonstrate indirect causal effects [48]. The observed relationships can guide selection among these accessions but require validation in independent environments.

### 4.4. Principal Component Analysis, Cluster Analysis, and Parental Selection

This study characterized the phenotypic structure of local peanut (*A. hypogaea*) germplasm from Guizhou. The accessions had many intercorrelated traits, so no single trait adequately represented overall variation. PCA reduced the traits to several relatively independent composite factors, mainly representing yield, kernel characteristics, sugars, amino acids, phenology, and fatty acids. This low-dimensional structure is consistent with previous germplasm studies [8,39] and indicates that integrating multiple traits provides a more comprehensive assessment than single-trait selection [49,50]. Using a composite score, we identified 137 accessions with F ≥ 0.00 and 34 with F ≥ 1.00 as priority candidates for core collection construction and hybrid parent selection [50,51]. Cluster analysis grouped the materials into four phenotypic classes—high-protein, early-maturing/high-yield, high-oleic, and large-kernel. These classes describe phenotypic similarity, not separable heritable components, and do not demonstrate that different quality traits are controlled by distinct genetic loci. Known high-oleic material is mainly governed by ahFAD2 alleles [52], and major-effect QTLs for protein content have been reported [53], but these findings cannot be directly inferred from the present cluster analysis. The apparent separation of high-oleic and high-sugar accessions should therefore be treated as a hypothesis for future genetic testing. Hybridization between representative accessions from Clusters II and III may combine early maturity, high yield, and high-oleic traits [29,33]. The lack of correspondence between clusters and geographic origin may reflect seed exchange and ecological convergence, but gene flow was not measured in this study [36]. Overall, PCA, composite scoring, and cluster analysis describe the phenotypic structure of the collection; selected accessions are candidates for further multi-environment and molecular evaluation.

### 4.5. GWAS Results for Protein and Sucrose

We analyzed 300 local peanut (*A. hypogaea*) germplasm accessions from Guizhou and conducted GWAS using three consecutive years of protein and sucrose measurements and 17,922 SNPs. The EMMAX mixed model used a kinship matrix estimated from the filtered SNPs and no additional principal-component covariates. Because the threshold −log10(P) > 3 was not corrected for multiple testing, we detected 43 protein-associated SNPs (33 above the suggestive threshold) and 41 sucrose-associated SNPs (27 above the suggestive threshold); these are candidate associations rather than genome-wide significant loci. Some associated chromosomal regions overlap those previously reported for peanut protein or sucrose/oil-related QTLs [29,53], but SNP-level overlap could not be established without harmonizing marker positions. The multi-locus, multi-chromosomal pattern is consistent with complex inheritance but does not by itself demonstrate polygenic control. These loci should be prioritized for validation, gene annotation, and potential marker development rather than being interpreted as direct targets.

## 5. Conclusions

We used 300 local peanut accessions from nine prefectures and cities in Guizhou, collected during the Third National Crop Germplasm Census, as study materials. Using the data for 30 traits recorded over three consecutive years—covering phenology, plant morphology, yield, and quality—we analyzed phenotypic diversity and breeding value. The germplasm showed wide phenotypic variation for most traits; the Shannon-Wiener diversity index and coefficient of variation indicated substantial phenotypic variation, particularly for flowering time, number of fruiting branches, and per-plant productivity. These materials provide a basis for breeding early-maturing, high-yielding, and specialty cultivars.

Regional differences were observed in phenotypic diversity patterns. Germplasm from Qianxinan and Qiandongnan had relatively high overall diversities, whereas Guiyang and Qiandongnan material showed extensive variation in yield-related traits. Bijie accessions had higher sucrose, total sugar, and fructose contents and may be promising for fresh-eating peanut breeding. Guiyang and Qiannan germplasm had higher arginine levels. These regional patterns may reflect ecological conditions, farmer selection, and seed exchange [36], but molecular data and multi-environment trials are needed to identify the underlying causes. Germplasm from high-diversity regions should be prioritized for conservation and further evaluation [8,36].

Trait correlations indicated statistical associations between quality traits, kernel development, plant architecture, and yield components. Protein and oil contents were negatively correlated, oleic acid was negatively correlated with linoleic and palmitic acids, and kernel size covaried with oil accumulation; these patterns are compatible with possible biological or metabolic explanations but should not be interpreted as demonstrated mechanisms. PCA reduced the correlated traits to relatively independent composite factors, including yield, kernel traits, sugars, amino acids, maturity duration, and fatty acids. Using a contribution-weighted composite score, 137 accessions with F ≥ 0.00 and 34 with F ≥ 1.00 were designated as priority candidates for core germplasm construction and parent selection. Cluster analysis divided the materials into four phenotypic groups—high-protein, early-maturing/high-yield, high-oleic, and large-kernel. The separation of high-oleic and high-sugar groups indicates phenotypic differentiation in this collection, but it does not demonstrate that oil and sucrose metabolism are regulated by different loci or pathways. Crosses between representative accessions are proposed as a breeding hypothesis rather than a demonstrated combination of traits.

The GWAS identified candidate SNP associations for protein and sucrose. We detected 43 protein-associated SNPs (33 above a suggestive threshold) and 41 sucrose-associated SNPs (27 above a suggestive threshold) across multiple chromosomes. Because the threshold was not corrected for multiple testing, these SNPs should be described as candidate loci for further validation, gene annotation, and potential marker development rather than direct targets [29]. The EMMAX model accounted for population structure through the kinship matrix, and multi-year phenotype means reduced the influence of year-to-year environmental noise, but independent populations and multi-location trials are still required to validate the associations. Candidate-gene functions should be assessed using reference-genome annotation [27].

In summary, the 300 Guizhou peanut germplasm accessions harbor substantial and exploitable phenotypic variation. PCA, comprehensive scoring, cluster analysis, and GWAS characterized the phenotypic structure and identified candidate SNP associations for protein and sucrose. We identified superior accessions that combine representativeness with desirable trait profiles; these can be used for parental selection in hybrid crosses, construction of core germplasm collections, and marker-assisted breeding. Subsequent multi-year, multi-location evaluations, whole-genome resequencing, and functional validation are needed to confirm the genetic basis of quality traits and support coordinated improvement of high protein, high sugar, high oleic acid, and high yield in Guizhou peanut breeding.

## Figures and Tables

**Figure 1:**
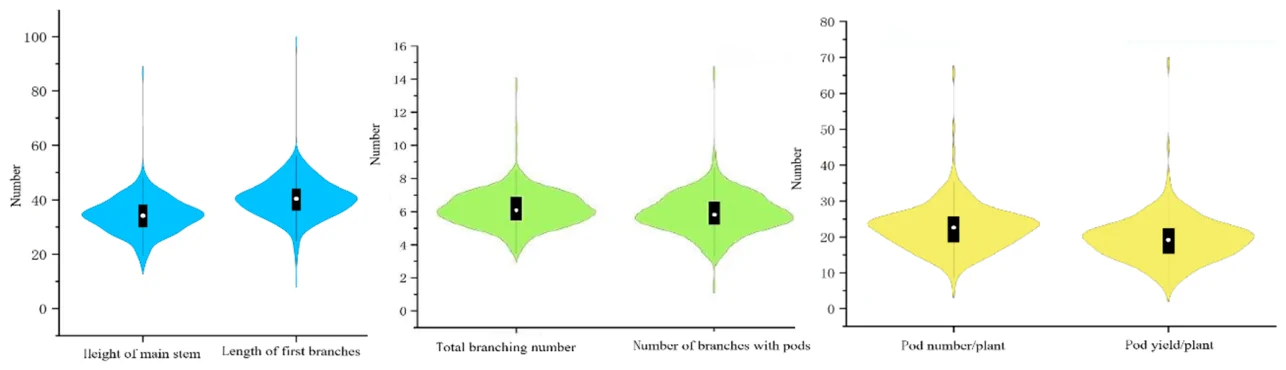
Distribution of six plant trait datasets of Guizhou local peanut germplasm resources.

**Figure 2:**
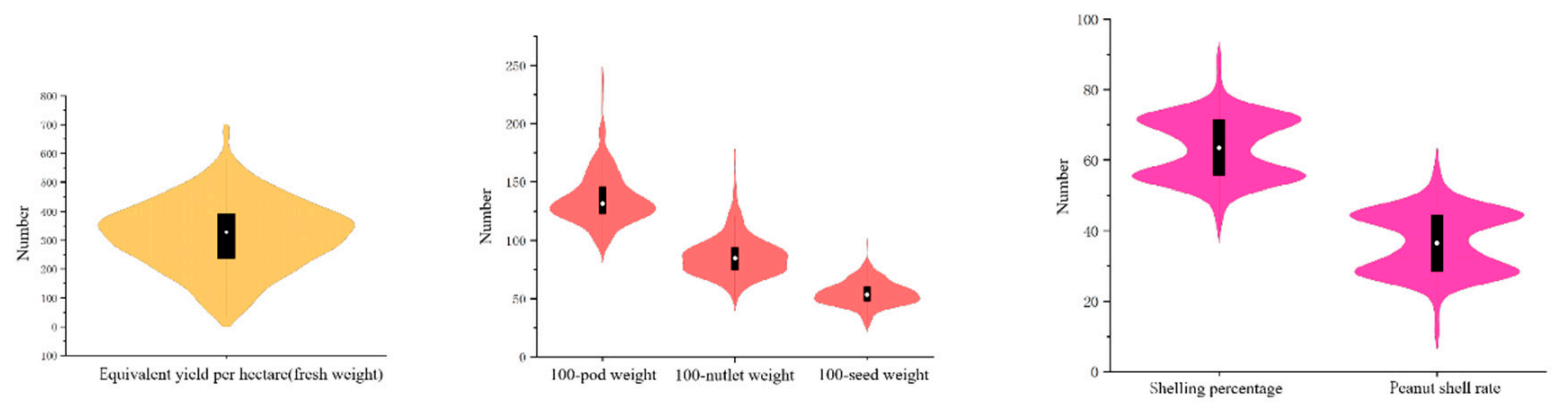
Distribution of six yield-related trait datasets of local peanut germplasm resources from Guizhou.

**Figure 3:**
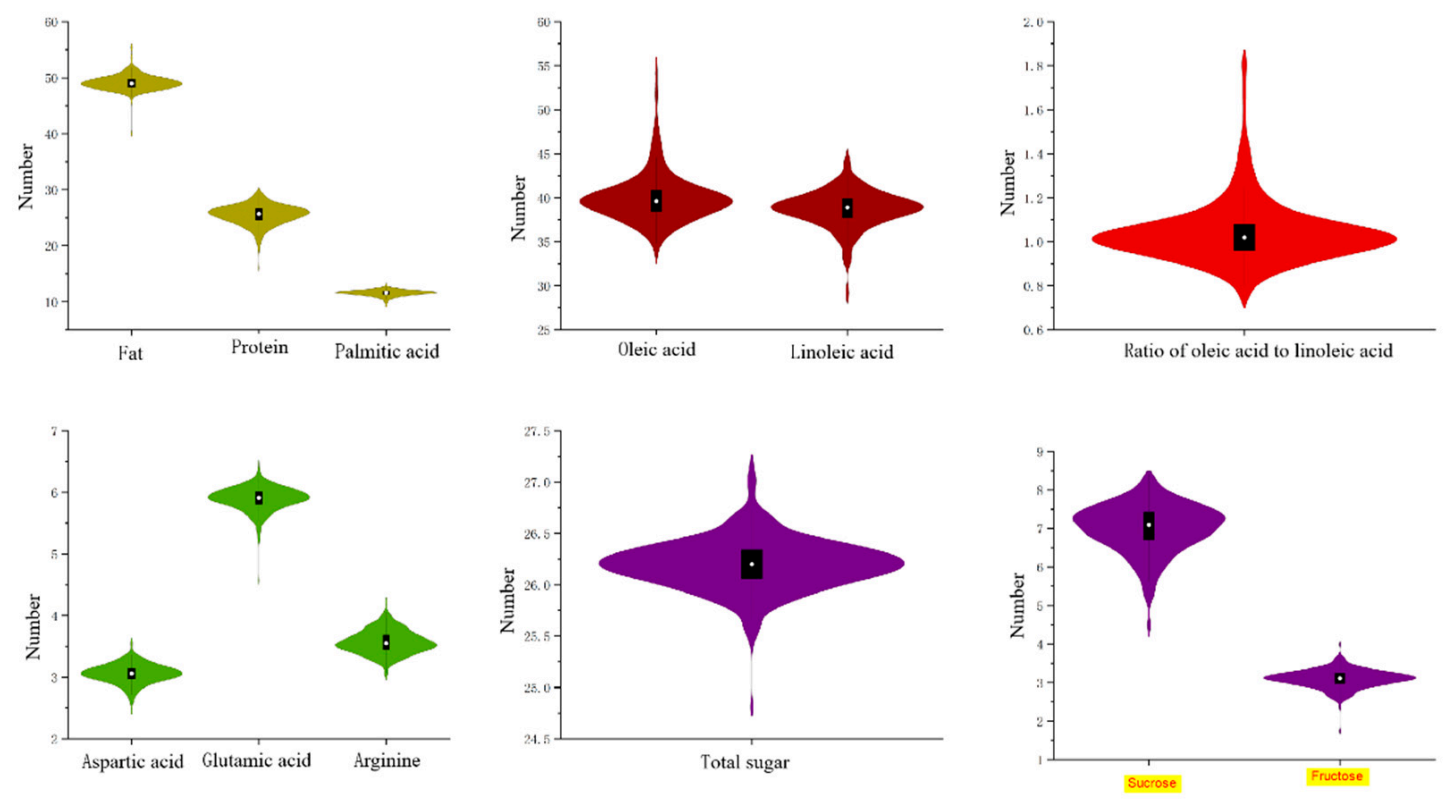
Distribution of twelve quality-related traits in local peanut germplasm from Guizhou.

**Figure 4:**
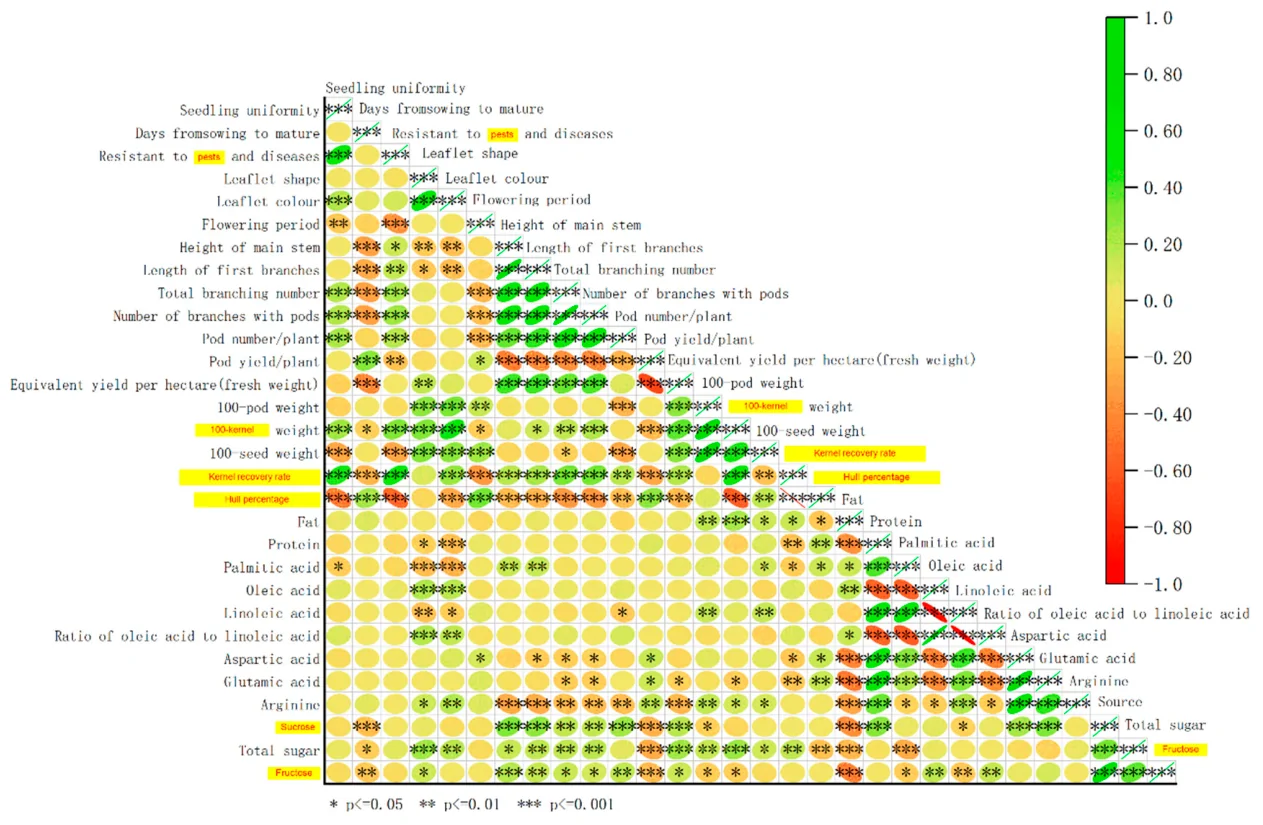
Correlation matrix of 30 phenotypic traits in Guizhou local peanut germplasm. The high-resolution figure preserves the original correlation values and highlights significant correlations.

**Figure 5:**
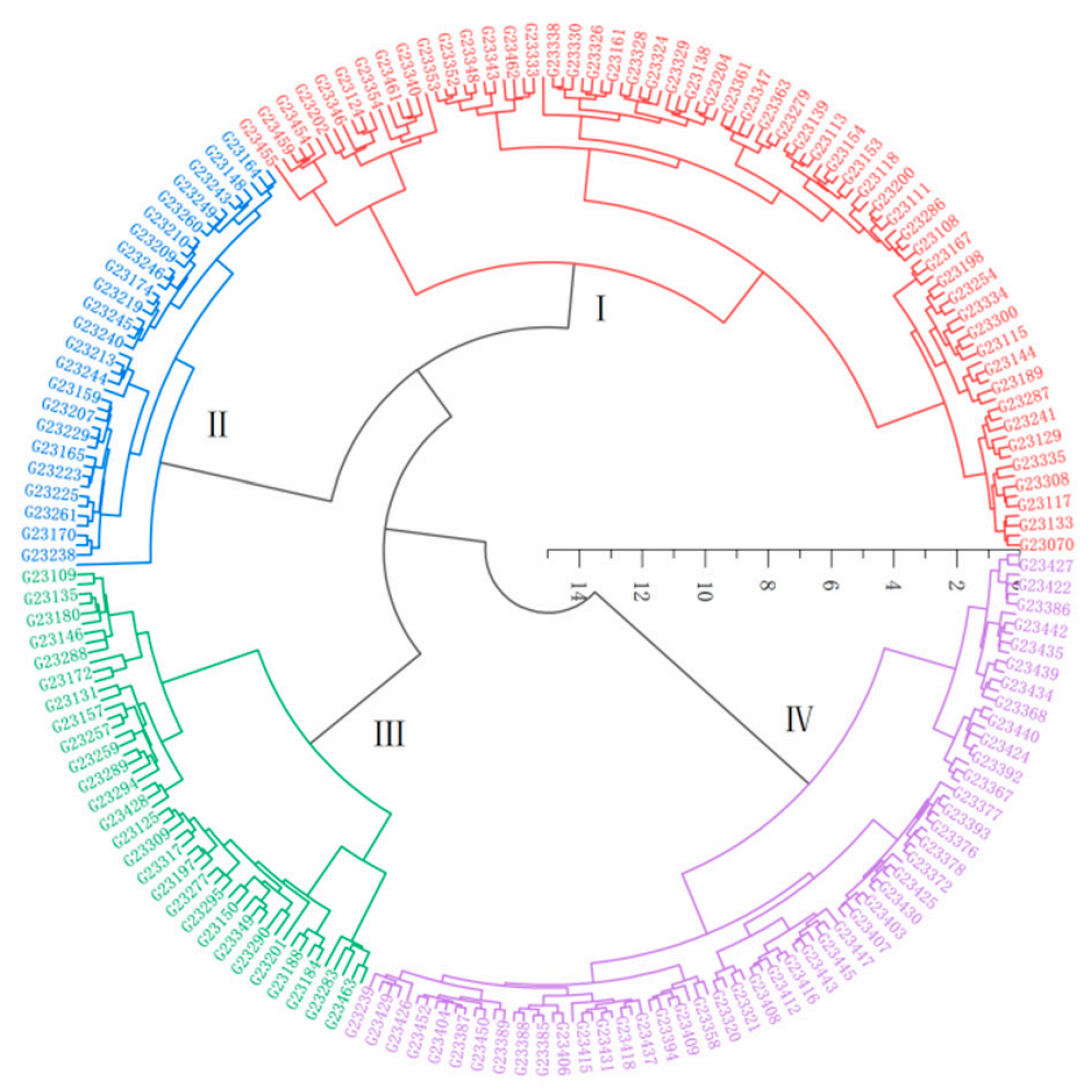
Cluster plot of Guizhou local peanut germplasm based on 30 phenotypic traits. Points are colored by group; visually overlapping points represent accessions with similar phenotypic profiles and do not alter the group assignments.

**Figure 6:**
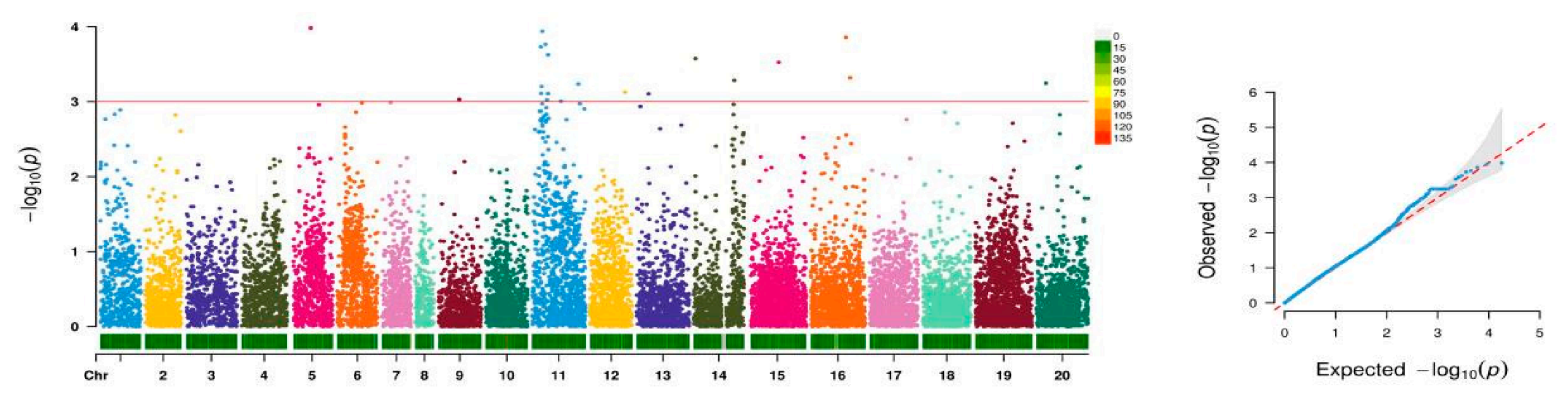
Manhattan and QQ plots for protein-associated SNPs in Guizhou local peanut germplasm. Horizontal lines indicate the suggestive and Bonferroni thresholds (−log10(P) = 3 and 5.55, respectively); the diagonal line in the QQ plot is the expected null line. The genomic inflation factor (λGC) was approximately 1.0.

**Figure 7:**
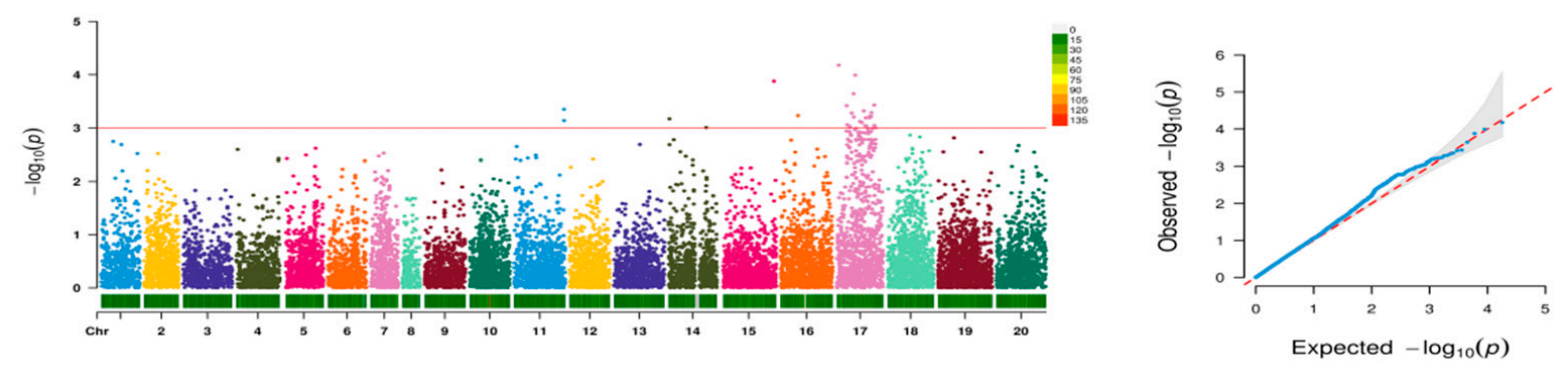
Manhattan and QQ plots for sucrose-associated SNPs in Guizhou local peanut germplasm. Horizontal lines indicate the suggestive and Bonferroni thresholds (−log10(P) = 3 and 5.55, respectively); the diagonal line in the QQ plot is the expected null line. The genomic inflation factor (λGC) was approximately 1.0.

**Table 1:** Assignment of phenotypic traits during the growth period.

**Traits**	0	1	2	3	4	5	6	7	8	9	10	11	12	**13**
Seedling uniformity	Uniformity	Little variation	Variation											
Resistant to pests and diseases	Disease-free plants	Diseased plant ≤ 1/3		1/3 > Diseased plant ≤ 1/2		1/2 > Diseased plant ≤ 1/3		Diseased plant ≥ 2/3		All plants exhibited symptoms of infection				
Leaflet shape		Cuneate	Obcuneate	Elliptic	Oblong-elliptic	Wide-elliptic	Suborbicular	Orbicular	Ovate	Obovate	Oblong	Oblong-lanceplate	Lanceolate	Linear-lanceolate
Leaflet color		Yellow-green	Light green	Green	Dark green	Bluish green								

**Table 2:** Frequency distribution and phenotypic diversity indices of 30 traits in local peanut germplasm resources from Guizhou.

Traits	H’	1 (%)	2 (%)	3 (%)	4 (%)	5 (%)	6 (%)	7 (%)	8 (%)	9 (%)	10 (%)	H’								
												Guiyang City	Liupoanshui City	Zunyi City	Tongren City	Southwest Guizhou Autonomous Prefecture	Bijie City	Anshun	Qiandongnan Miao and Dong Autonomous Prefecture	Buyi and Miao Autonomous Prefecture of QianNan
Seedling uniformity	0.68	0.00	0.45	0.55	0.00							0.52	0.69	0.55	0.69	0.63	0.46	0.67	0.63	0.68
Days from sowing to maturity	0.85	0.00	0.42	0.53	0.05							0.63	0.69	0.87	0.85	0.80	0.35	0.87	0.86	0.73
Resistant to pests and diseases	0.88	0.00	0.1	0.66	0.24	0.01	0.00					0.88	0.97	0.89	0.56	1.02	0.53	0.63	1.03	0.63
Leaflet shape	0.98	0.00	0.68	0.19	0.07	0.02	0.02	0.01	0.00			0.98	0.56	0.90	0.82	1.13	0.53	0.80	1.00	0.95
Leaflet color	0.54	0.00	0.00	0.05	0.86	0.07	0.02					0.67	0.00	0.37	0.42	0.85	0.35	0.28	0.56	0.30
Flowering period	1.31	0.01	0.18	0.00	0.16	0.08	0.02	0.53	0.00	0.00	0.01	1.08	0.69	1.41	1.49	1.31	1.21	1.10	0.99	0.82
Height of main stem	1.98	0.03	0.02	0.08	0.14	0.22	0.23	0.14	0.10	0.02	0.01	1.74	1.67	2.07	1.70	1.88	1.74	1.76	2.05	1.90
Length of first branches	1.96	0.01	0.04	0.08	0.16	0.21	0.25	0.12	0.09	0.04	0.01	1.41	1.21	1.98	1.60	1.94	1.68	1.93	1.79	1.98
Total branching number	2.00	0.02	0.03	0.08	0.17	0.22	0.20	0.15	0.08	0.03	0.02	1.46	1.56	1.98	1.73	2.05	1.74	1.91	1.99	1.78
Number of branches with pods	2.01	0.02	0.03	0.09	0.16	0.24	0.18	0.14	0.09	0.03	0.02	1.67	1.73	2.03	1.82	1.96	1.89	1.99	2.00	1.82
Pod number per plant	1.98	0.01	0.03	0.11	0.16	0.19	0.25	0.13	0.06	0.04	0.02	1.59	1.73	2.04	1.74	1.96	1.30	1.93	1.91	2.00
Pod yield per plant	1.96	0.01	0.05	0.07	0.17	0.21	0.25	0.12	0.08	0.02	0.02	1.56	1.49	1.94	1.90	1.89	1.74	1.89	2.07	1.97
Equivalent yield per hectare (fresh weight)	2.09	0.03	0.05	0.09	0.12	0.19	0.21	0.15	0.10	0.04	0.02	1.93	1.49	1.96	2.02	2.04	1.15	1.74	1.96	1.98
100-pod weight	1.94	0.00	0.04	0.06	0.21	0.30	0.15	0.10	0.06	0.04	0.04	1.79	1.39	1.96	1.53	1.90	1.43	1.77	1.99	1.94
100-kernel weight	1.98	0.01	0.03	0.08	0.21	0.22	0.21	0.12	0.04	0.03	0.04	1.87	0.97	1.92	1.68	1.96	1.74	1.68	1.90	2.02
100-seed weight	2.03	0.02	0.02	0.07	0.21	0.20	0.19	0.12	0.07	0.05	0.03	1.96	1.56	2.06	1.88	2.03	1.74	1.95	1.86	1.94
Kernel recovery rate	1.92	0.01	0.02	0.12	0.27	0.07	0.11	0.21	0.15	0.01	0.02	1.73	0.66	1.80	1.86	1.66	1.68	1.55	1.77	1.80
Hull percentage	1.92	0.02	0.01	0.15	0.21	0.11	0.07	0.27	0.12	0.02	0.01	1.73	0.66	1.74	1.86	1.66	1.68	1.55	1.77	1.80
Fat	1.92	0.01	0.01	0.09	0.18	0.25	0.20	0.15	0.04	0.05	0.02	1.42	1.07	1.88	1.84	2.06	1.46	2.04	1.76	2.03
Protein	2.01	0.04	0.03	0.07	0.12	0.17	0.24	0.20	0.07	0.04	0.01	1.93	1.73	1.80	1.76	1.77	1.74	2.00	2.03	2.01
Palmitic acid	2.01	0.04	0.04	0.06	0.12	0.19	0.26	0.17	0.08	0.04	0.01	1.80	1.73	1.96	1.91	1.75	1.43	2.04	1.88	2.00
Oleic acid	1.84	0.00	0.01	0.08	0.18	0.31	0.22	0.08	0.04	0.02	0.04	1.24	1.73	1.91	1.59	1.54	1.43	1.59	1.82	1.78
Linoleic acid	1.96	0.04	0.03	0.05	0.10	0.24	0.27	0.15	0.08	0.02	0.02	1.81	1.67	2.01	1.89	1.62	1.18	1.91	1.94	1.76
Ratio of oleic acid to linoleic acid	1.81	0.00	0.02	0.07	0.19	0.31	0.23	0.08	0.04	0.02	0.05	1.35	1.49	1.98	1.70	1.27	1.21	1.78	1.83	1.71
Aspartic acid	1.92	0.03	0.04	0.06	0.14	0.21	0.22	0.15	0.09	0.05	0.01	1.65	1.49	2.02	1.97	1.94	1.68	1.94	1.93	1.84
Glutamic acid	1.95	0.03	0.04	0.06	0.10	0.22	0.25	0.19	0.08	0.02	0.01	1.85	1.67	2.02	1.92	1.41	1.74	1.81	2.04	1.78
Arginine	2.07	0.02	0.04	0.10	0.15	0.24	0.16	0.13	0.09	0.05	0.03	1.90	1.32	1.99	2.03	2.05	1.68	1.93	1.82	1.97
Sucrose	1.99	0.04	0.03	0.07	0.10	0.20	0.23	0.19	0.11	0.03	0.00	1.99	1.26	1.89	1.98	1.86	1.74	1.74	1.98	1.90
Total sugar	2.00	0.03	0.02	0.08	0.14	0.22	0.24	0.15	0.06	0.04	0.02	1.81	1.56	1.83	1.81	2.00	1.52	1.96	1.91	1.82
Fructose	2.01	0.02	0.05	0.06	0.12	0.21	0.26	0.15	0.07	0.03	0.02	1.81	1.21	1.84	1.96	1.99	1.58	1.95	1.92	1.82

**Table 3:** Mean values and coefficients of variation of phenotypic traits of Guizhou local peanut germplasm resources.

Traits	Max	Min	Average	SD	CV/%
Height of main stem	86.78	16.19	34.19	6.89	20.15
Length of first branches	98.78	10.29	40.33	7.63	18.91
Total branching number	13.67	3.46	6.16	1.10	17.86
Number of branches with pods	14.33	1.50	5.91	1.22	20.68
Pod number per plant	65.64	5.26	22.50	6.38	28.36
Pod yield per plant	68.54	4.76	19.30	6.32	32.75
Equivalent yield per hectare (fresh weight)	682.66	31.68	317.86	118.98	37.43
100-pod weight	241.24	90.98	135.47	21.87	16.14
100-kernel weight	172.06	47.29	86.37	17.30	20.03
100-seed weight	97.52	25.90	54.29	10.34	19.04
Kernel recovery rate	89.44	39.80	63.29	8.83	13.96
hull percentage	60.20	10.56	36.71	8.83	24.07
Fat	55.52	39.96	49.12	1.37	2.78
Protein	29.51	16.08	25.51	1.81	7.09
Palmitic acid	13.18	9.30	11.58	0.55	4.72
Oleic acid	55.17	33.65	40.05	2.95	7.36
Linoleic acid	44.68	28.79	38.69	2.18	5.64
Ratio of oleic acid to linoleic acid	1.82	0.77	1.04	0.14	13.85
Aspartic acid	3.57	2.44	3.06	0.16	5.20
Glutamic acid	6.47	4.57	5.89	0.20	3.34
Arginine	4.23	3.04	3.57	0.19	5.36
Sucrose	8.41	4.48	7.00	0.64	9.18
Total sugar	27.18	24.81	26.20	0.26	0.99
Fructose	3.99	1.74	3.10	0.24	7.71

**Table 4:** Screening of influential factors and construction of regression equations for the effects of whole-life reproductive traits and agronomic traits on quality traits of Guizhou local peanut germplasm resources.

Traits	Main Influencing Factor	Regression Equation	*p*
Fat/Y1	100-kernel weight/X15	Y1 = 47.554 + 0.018X15	0.000
Protein/Y2	Leaf color/X5	Y2 = 28.047 − 0.794X5	0.001
Palmitic acid/Y3	Leaf color/X5, 100-kernel weight/X16	Y3 = 12.213 − 0.382X5 + 0.011X16	0.000
Oleic acid/Y4	Leaf shape/X4, 100-kernel weight/X16	Y4 = 39.472 + 0.688X4 − 0.050X16	0.003
Linoleic acid/Y5	Leaf shape/X4, 100-kernel weight/X16	Y5 = 38.272 − 0.475X4 + 0.050X16	0.000
Ratio of oleic acid to linoleic acid/Y6	Leaf shape/X4, 100-kernel weight/X16	Y6 = 1.029 + 0.034X4 − 0.003X16	0.000
Aspartic acid/Y7	/	/	/
Glutamic acid/Y8	Hull percentage/X18, Resistant to pests and diseases/X3	Y8 = 5.629 + 0.005X18 + 0.032X3	0.001
Arginine/Y9	Length of first branches/X8, 100-pod weight/X14	Y9 = 3.585 − 0.005X8 + 0.002X14	0.000
Sucrose/Y10	Height of main stem/X7, Pod number per plant/X11, Leaf shape/X4, 100-pod weight/X14, Pod yield per plant/X12	Y10 = 6.226 + 0.023X7 + 0.013X11 + 0.090X4 − 0.007X14 − 0.001X12	0.000
Total sugar/Y11	Leaf shape/X4, 100-pod weight/X14	Y11 = 25.890 + 0.044X4 + 0.002X14	0.000
Fructose/Y12	100-pod weight/X14, Leaf shape/X4, Height of main stem/X7	Y12 = 3.014 − 0.003X14 + 0.046X4 + 0.006X7	0.000

**Table 5:** Principal component analysis of 30 phenotypic traits of 300 Guizhou peanut germplasm resources.

Traits	PC1	PC2	PC3	PC4	PC5	PC6	PC7	PC8
Seedling uniformity	0.15	0.00	0.06	−0.23	0.28	0.08	0.00	−0.09
Days from sowing to maturity	−0.14	−0.15	0.00	−0.10	0.06	0.09	0.56	0.28
Resistant to pests and diseases	0.18	0.07	0.06	−0.24	0.26	−0.07	−0.01	0.25
Leaf shape	0.06	−0.08	0.29	0.23	0.09	0.09	0.18	−0.24
Leaf color	0.08	−0.09	0.34	0.10	0.18	0.16	0.14	−0.19
Flowering period	−0.14	−0.03	0.03	0.18	−0.15	0.12	−0.41	−0.26
Height of main stem	0.22	0.23	−0.14	0.07	−0.17	0.09	−0.07	0.26
Length of first branches	0.24	0.23	−0.13	0.04	−0.17	0.15	−0.02	0.27
Total branching number	0.28	0.18	−0.06	0.00	−0.03	0.31	0.11	−0.27
Number of branches with pods	0.29	0.20	−0.05	−0.01	−0.06	0.33	0.12	−0.23
Pod number per plant	0.20	0.13	−0.19	−0.01	0.08	0.34	0.26	−0.07
Pod yield per plant	−0.23	−0.16	−0.02	−0.11	0.13	0.20	0.10	0.08
Equivalent yield per hectare (fresh weight)	0.20	0.17	0.14	0.17	−0.25	−0.12	−0.19	−0.07
100-pod weight	−0.02	0.01	0.42	0.13	−0.20	0.15	0.08	0.26
100-kernel weight	0.17	0.04	0.45	−0.05	−0.01	0.00	−0.01	0.21
100-seed weight	−0.07	0.01	0.36	0.21	−0.26	0.07	0.05	0.11
Kernel recovery rate	0.28	0.07	0.17	−0.23	0.20	−0.20	−0.16	0.02
Hull percentage	−0.28	−0.07	−0.17	0.23	−0.20	0.20	0.16	−0.02
Fat	0.09	−0.16	0.10	−0.27	−0.29	0.09	0.01	0.15
Protein	−0.20	0.30	0.01	0.03	0.09	0.08	0.03	0.10
Palmitic acid	−0.13	0.26	−0.01	−0.16	−0.21	−0.01	0.04	0.16
Oleic acid	0.20	−0.34	−0.06	0.19	0.08	0.13	−0.14	0.20
Linoleic acid	−0.20	0.32	0.13	−0.19	−0.09	−0.14	0.15	−0.15
Ratio of oleic acid to linoleic acid	0.20	−0.34	−0.08	0.18	0.09	0.16	−0.14	0.18
Aspartic acid	−0.21	0.25	0.10	0.09	0.26	0.21	−0.17	0.08
Glutamic acid	−0.19	0.24	0.00	0.10	0.28	0.18	−0.15	0.13
Arginine	−0.15	0.07	0.19	0.08	0.31	0.19	−0.17	0.03
Sucrose	0.07	0.19	−0.13	0.36	0.15	−0.16	0.07	0.28
Total sugar	0.13	0.07	0.11	0.25	0.08	−0.35	0.32	−0.14
Fructose	0.08	0.09	−0.14	0.39	0.16	−0.28	0.13	0.04
Characteristic value	5.72	4.47	3.43	2.87	2.75	1.31	1.15	1.03
Contribution rate%	19.07	14.89	11.43	9.58	9.16	4.37	3.83	3.44
Cumulative contribution rate%	19.07	33.96	45.39	54.97	64.12	68.49	72.32	75.77

**Table 6:** Comprehensive scores of 30 phenotypic traits for 300 local peanut germplasm accessions from Guizhou.

Serial Number	Comprehensive Score (F)	Serial Number	Comprehensive Score (F)	Serial Number	Comprehensive Score (F)	Serial Number	Comprehensive Score (F)
G23070	0.49	G23200	−0.09	G23309	−0.81	G23390	−0.12
G23106	0.06	G23201	−1.48	G23316	0.85	G23391	−0.52
G23107	1.08	G23202	−0.27	G23317	−0.17	G23392	−0.18
G23108	−0.25	G23203	0.81	G23318	1.31	G23393	−0.12
G23109	1.51	G23204	−0.11	G23319	−0.47	G23394	−0.17
G23110	0.74	G23206	0.39	G23320	−0.39	G23395	−0.10
G23111	0.09	G23207	0.26	G23321	−0.66	G23396	−0.31
G23112	0.60	G23208	0.95	G23322	−0.76	G23397	−0.17
G23113	0.90	G23209	1.14	G23323	−0.37	G23398	−0.93
G23114	−0.02	G23210	0.87	G23324	−0.65	G23399	−0.44
G23115	−0.41	G23211	0.74	G23325	−0.64	G23400	0.28
G23116	−0.52	G23212	4.05	G23326	0.30	G23401	0.02
G23117	0.08	G23213	0.68	G23327	0.04	G23402	−0.12
G23118	−0.59	G23214	0.58	G23328	−0.77	G23403	0.15
G23119	−0.07	G23215	1.12	G23329	0.28	G23404	−0.82
G23120	0.01	G23217	1.10	G23330	−0.08	G23405	−0.18
G23121	0.41	G23219	1.23	G23331	0.22	G23406	0.00
G23122	0.34	G23223	0.46	G23332	0.39	G23407	−0.13
G23123	−0.54	G23224	0.56	G23333	−0.93	G23408	−0.95
G23124	−0.89	G23225	1.34	G23334	0.15	G23409	−0.06
G23125	−0.20	G23226	1.05	G23335	−0.40	G23410	−0.31
G23126	−0.65	G23228	0.69	G23336	−0.33	G23411	−0.59
G23127	0.81	G23229	0.57	G23337	−0.21	G23412	−0.41
G23128	0.81	G23230	0.64	G23338	0.00	G23413	−1.01
G23129	0.75	G23233	1.06	G23339	0.23	G23414	−0.78
G23130	0.51	G23237	1.00	G23340	−1.87	G23415	−0.71
G23131	1.63	G23238	0.56	G23341	−1.06	G23416	−0.07
G23132	0.90	G23239	−0.42	G23342	−0.24	G23417	−0.22
G23133	0.85	G23240	1.33	G23343	0.35	G23418	−1.00
G23134	−0.73	G23241	1.01	G23344	−0.07	G23419	−1.11
G23135	0.78	G23243	0.28	G23345	−0.92	G23420	−0.29
G23136	0.25	G23244	0.86	G23346	−0.94	G23421	−0.66
G23137	0.72	G23245	0.81	G23347	−0.45	G23422	−0.50
G23138	0.27	G23246	0.96	G23348	−0.76	G23423	−0.30
G23139	1.09	G23247	0.14	G23349	−0.61	G23424	−0.19
G23140	−0.54	G23249	0.84	G23350	−1.57	G23425	−0.45
G23141	−0.14	G23250	0.86	G23351	−0.76	G23426	−1.07
G23144	0.17	G23251	0.41	G23352	−0.35	G23427	−1.68
G23145	0.47	G23252	0.46	G23353	−0.05	G23428	−0.31
G23146	0.07	G23253	1.93	G23354	−2.19	G23429	−1.12
G23147	−0.86	G23254	0.60	G23355	0.00	G23430	−0.14
G23148	−0.35	G23255	0.47	G23356	0.66	G23431	−0.72
G23149	−0.15	G23256	0.57	G23357	−0.37	G23432	−1.44
G23150	−0.28	G23257	1.30	G23358	−0.63	G23433	−0.22
G23151	−0.86	G23258	0.82	G23359	0.95	G23434	−0.64
G23152	0.76	G23259	0.78	G23360	−0.05	G23435	−0.09
G23153	−0.01	G23260	0.43	G23361	0.37	G23436	−0.39
G23154	0.36	G23261	1.02	G23362	−0.13	G23437	−1.30
G23156	0.30	G23262	0.82	G23363	−0.45	G23438	−0.64
G23157	1.07	G23263	1.35	G23364	−0.59	G23439	−0.71
G23158	0.54	G23275	−0.04	G23365	−0.86	G23440	−0.25
G23159	0.34	G23277	−0.18	G23366	0.32	G23441	−0.34
G23161	0.73	G23278	1.03	G23367	0.35	G23442	−0.28
G23163	−0.08	G23279	−0.05	G23368	−0.31	G23443	−0.67
G23164	0.61	G23280	1.33	G23369	0.11	G23444	−0.50
G23165	0.59	G23281	0.06	G23370	0.09	G23445	−0.47
G23166	1.20	G23282	0.12	G23371	−0.07	G23446	−1.23
G23167	1.10	G23283	−0.48	G23372	0.14	G23447	−0.19
G23169	2.60	G23284	0.43	G23373	0.26	G23448	−0.93
G23170	1.12	G23285	−0.06	G23374	0.10	G23449	−0.52
G23171	1.26	G23286	−0.13	G23375	−0.13	G23450	−1.49
G23172	1.36	G23287	−0.13	G23376	−0.19	G23451	−0.90
G23174	1.48	G23288	0.74	G23377	0.48	G23452	−0.69
G23176	0.52	G23289	1.95	G23378	0.29	G23453	−0.54
G23177	0.07	G23290	0.05	G23379	−0.21	G23454	−1.40
G23178	0.96	G23291	0.58	G23380	−0.47	G23455	−3.00
G23180	1.33	G23292	−0.59	G23381	0.46	G23456	−1.35
G23182	0.73	G23294	1.72	G23382	−0.34	G23457	−1.96
G23184	0.51	G23295	−0.56	G23383	−0.49	G23458	−2.43
G23186	2.06	G23297	−0.13	G23384	−0.91	G23459	−1.00
G23188	−0.54	G23298	−0.49	G23385	−0.32	G23460	−2.31
G23189	0.75	G23300	−0.14	G23386	−0.33	G23461	−1.92
G23196	0.07	G23301	−0.31	G23387	−0.61	G23462	−1.43
G23197	−0.13	G23302	0.42	G23388	−0.46	G23463	−1.91
G23198	0.14	G23308	−0.57	G23389	−0.45	G23464	−1.46

**Table 7:** Characteristics of different groups of whole-growth-period traits of germplasm resources from various origins in Guizhou.

Traits	Group I (n = 105)		Group II (*n* = 47)		Group III (*n* = 54)		Group IV (*n* = 94)	
	Mean	CV/%	Mean	CV/%	Mean	CV/%	Mean	CV/%
Seedling uniformity	1.98	19.85	2.16	15.41	2.08	19.92	1.43	35.43
The entire growth period	2.13	20.30	1.06	21.13	1.86	32.31	1.72	23.15
Resistant to pests and diseases	2.44	44.60	2.58	24.60	2.22	38.10	0.98	43.48
Leaf shape	4.54	18.28	4.35	16.46	5.73	33.00	4.79	15.17
Leaf color	3.18	12.42	3.06	7.04	3.61	19.21	3.04	7.23
Flowering period	38.77	11.46	38.63	8.55	40.56	9.26	42.90	4.73

**Table 8:** Representative materials of different peanut germplasm groups.

Group	Main Characteristic	Typical Materials of Different Groups of Peanut Germplasm
Group I	days from sowing to maturity long, pod yield per plant high, protein, palmitic acid, linoleic acid, aspartic acid, glutamic acid, arginine high.	Xishui niba peanut, Mannai Peanut, Luodong Peanut (Red), Wuyá Peanut, Mukoong Peanut
Group II	seedling uniformity, days from sowing to maturity short, resistance to pests and diseases strong, flowering period short, height of main stem high, length of first branches long, total branching number, number of branches with pods, pod number per plant more, equivalent yield/hectare (fresh weight), kernel recovery rate high, hull percentage low, source, fructose high.	Xingyi small peanut, Dejiang Longquan peanut, Longtan peanut, Shangba peanut, Chilunchang small peanut, Chishui City Yuanhou Town peanut
Group III	100-pod weight, kernel recovery rate, fat, oleic acid, ratio of oleic acid to linoleic acid, total sugar high.	Bianyang peanut, Woyan duoli peanut, Songtao Shichang peanut, Chishui Fuxing-1, Shiqian Ganxi Town peanut, Guiyang Duoli Red
Group IV	flowering period long, 100-seed weight, hull percentage high.	Yaoshang peanut, Bantun peanut (3 seeds), Qiujiami peanut, Houchangba peanut, Kaiyang peanut, Liuchang peanut

## Data Availability

All data generated or analyzed during this study are included in this published article. The raw phenotypic datasets are available from the corresponding author upon reasonable request.

## Supplementary Materials

The following supporting information can be downloaded at: https://www.mdpi.com/article/10.3390/genes17101164/s1, Table S1. Model-fit and residual diagnostics for the stepwise regression models of quality traits.

## Abbreviations

The following abbreviations are used in this manuscript:

QTL	Quantitative trait locus
GWAS	Genome-wide association study
SSR	Simple sequence repeat
SNP	Single nucleotide polymorphism
NIRS	Near-infrared spectroscopy
H’	Shannon-Wiener diversity index
CV	Coefficient of variation
FAO	Food and Agriculture Organization
MAS	Marker-assisted selection
PCA	Principal component analysis
