# Supplementary material for: Phenotypic Diversity Analysis, Comprehensive Evaluation, and GWAS of Protein and Sucrose Content in Guizhou Local Peanut Germplasm Based on Three Consecutive Years of Phenotypic Data
Source: Genes (Basel). 2026 Sep 22;17(10):1164. doi: 10.3390/genes17101164 (PMC13704605; doi:10.3390/genes17101164)
Supplement: Supplementary file 1 [file genes-17-01164-s001.zip › genes-4533739-supplementary.pdf]

Supplementary Materials

Table S1. Model-fit statistics, standardized coefficients, variance inflation factors, and residual diagnostics for the stepwise regression models.

Trait	Predictors retained	Model P	Adjusted R2	Standardized coefficients (beta, P, VIF)	Durbin-Watson
Fat	100-kernel weight	0.000	0.053	100-kernel weight: beta=0.229, P=0.000, VIF=1.00	DW=1.857
Protein	Leaf colour	0.001	0.039	Leaf colour: beta=-0.198, P=0.001, VIF=1.00	DW=1.766
Palmitic acid	Leaf colour; 100-seed weight	0.000	0.098	Leaf colour: beta=-0.315, P=0.000, VIF=1.09; 100-seed weight: beta=0.204, P=0.000, VIF=1.09	DW=1.635
Oleic acid	Leaf shape; 100-seed weight	0.000	0.066	Leaf shape: beta=0.269, P=0.000, VIF=1.13; 100-seed weight: beta=-0.177, P=0.003, VIF=1.13	DW=1.677
Linoleic acid	Leaf shape; 100-seed weight	0.000	0.073	Leaf shape: beta=-0.251, P=0.000, VIF=1.13; 100-seed weight: beta=0.237, P=0.000, VIF=1.13	DW=1.714
Oleic/linoleic acid ratio	Leaf shape; 100-seed weight	0.000	0.070	Leaf shape: beta=0.270, P=0.000, VIF=1.13; 100-seed weight: beta=-0.197, P=0.001, VIF=1.13	DW=1.595
Aspartic acid	None retained	-	0.000	No predictor met the entry criterion	DW=1.752
Glutamic acid	Hull percentage; resistance to pests and diseases	0.001	0.037	Hull percentage: beta=0.247, P=0.000, VIF=1.45; resistance: beta=0.169, P=0.014, VIF=1.45	DW=1.684
Arginine	Length of first branches; 100-pod weight	0.000	0.099	Length of first branches: beta=-0.271, P=0.000, VIF=1.00; 100-pod weight: beta=0.174, P=0.002, VIF=1.00	DW=2.138
Sucrose	Main stem height; pod number per plant; leaf shape; 100-pod weight; pod yield per plant	0.000	0.161	Main stem height: beta=0.250, P=0.000, VIF=1.44; pod number per plant: beta=0.128, P=0.028, VIF=1.18; leaf shape: beta=0.162, P=0.005, VIF=1.18; 100-pod weight: beta=-0.191, P=0.001, VIF=1.19; pod yield per plant: beta=-0.141, P=0.021, VIF=1.32	DW=1.446
Total sugar	Leaf shape; 100-pod weight	0.000	0.068	Leaf shape: beta=0.197, P=0.001, VIF=1.13; 100-pod weight: beta=0.138, P=0.019, VIF=1.17	DW=1.829
Fructose	100-pod weight; leaf shape; main stem height	1.05E-06	0.089	100-pod weight: beta=-0.248, P=0.000, VIF=1.17; leaf shape: beta=0.222, P=0.000, VIF=1.18; main stem height: beta=0.180, P=0.004, VIF=1.31	DW=1.770

Notes: Stepwise regression used an entry threshold of P<0.05 and a removal threshold of P>0.10. beta, standardized regression coefficient; VIF, variance inflation factor; DW, Durbin-Watson statistic; BP, Breusch-Pagan test; JB, Jarque-Bera test. Residual diagnostics with P<0.05 indicate departure from the tested assumption. Variables with P>0.05 in the final model were retained in the original stepwise solution but should be interpreted cautiously.
